# Transactions of the American Surgical Association

**Published:** 1892-12

**Authors:** 


*Transactions of the American Surgical Association.* Vol. IX. Edited by J. EWING MEARS, M.D., Recorder of the Association. Pp. xxviii., 508. Philadelphia: WILLIAM J. DORNAN. 1891.

The present volume fully maintains the high standard of its predecessors, and is a mine of wealth to the student of surgery. Most of the articles have appeared before in the weekly journals of New York, and many of them have been reproduced in this country; but they are all worthy of careful reading, and one or two will prove of lasting value to surgical literature.

There can be little doubt that the surgery of the other side of the Atlantic is at the present time of a very high order, and if English surgeons are to maintain their former reputation they will have to take hints from their American cousins. It is stated that the latter are now paying much greater attention to Germany than to this country for their ideas, and it is certain that both in Germany and America surgical *technique* is more fully studied and better carried out than with us.

There are 17 original papers in the volume before us, among which we would particularly mention: "The Present Status of Brain Surgery," by Dr. Hayes Agnew; "Aseptic and Antiseptic Details in Operative Surgery," by Dr. Arpad G. Gerster; and others by such well-known surgeons as Lewis A. Stimson, Stephen Smith, and Nicholas Senn.

The printing and binding are up to the usual high standard of American excellence.

